# Crystal structure of 4-[(*E*)-(4-hy­droxy­benzyl­idene)amino]-1,5-dimethyl-2-phenyl-1*H*-pyrazol-3(2*H*)-one

**DOI:** 10.1107/S2056989015021325

**Published:** 2015-11-14

**Authors:** Joel T. Mague, Mehmet Akkurt, Shaaban K. Mohamed, Alaa F. Mohamed, Mustafa R. Albayati

**Affiliations:** a Department of Chemistry, Tulane University, New Orleans, LA 70118, USA; b Department of Physics, Faculty of Sciences, Erciyes University, 38039 Kayseri, Turkey; c Chemistry and Environmental Division, Manchester Metropolitan University, Manchester M1 5GD, England; d Chemistry Department, Faculty of Science, Minia University, 61519 El-Minia, Egypt; e National Organization for Drug Control and Research, Giza, Egypt; f Kirkuk University, College of Science, Department of Chemistry, Kirkuk, Iraq

**Keywords:** crystal structure, pyrazolo­nes, bio-active motifs, hydrogen bonding

## Abstract

The asymmetric unit of the title compound, C_18_H_17_N_3_O_2_, comprises three independent mol­ecules (1, 2 and 3). In mol­ecule 1, the dihedral angles between the pyrazolone ring and the pendant phenyl and hydroxybenzene rings are 54.43 (6) and 28.72 (6)°, respectively. The corresponding data for mol­ecule 2 are 86.84 (6) and 25.69 (5)°, respectively, and for mol­ecule 3 are 47.41 (7) and 17.09 (7)°, respectively. The three mol­ecules feature an intra­molecular C—H⋯O inter­action, which closes an *S*(6) ring in each case. In the crystal, mol­ecules are linked by O—H⋯O hydrogen bonds, which generate [100] chains incorporating all three asymmetric mol­ecules. Two weak C—H⋯O interactions connect three independent molecules to each other along the c-axis direction.

## Related literature   

For the biological activities of the pyrazolone ring system, see: Nirali & Maulik (2010 ▸); Rahat *et al.* (2008 ▸); Thakkar & Joshi (2010 ▸); Mahmoud *et al.* (2011 ▸); Tripathy *et al.* (2007 ▸); Brune (1997 ▸); Abdel-Aziz *et al.* (2009 ▸). For industrial applications, see: Karci & Ertan (2002 ▸); Khalil *et al.* (2005 ▸); Ho (2005 ▸).
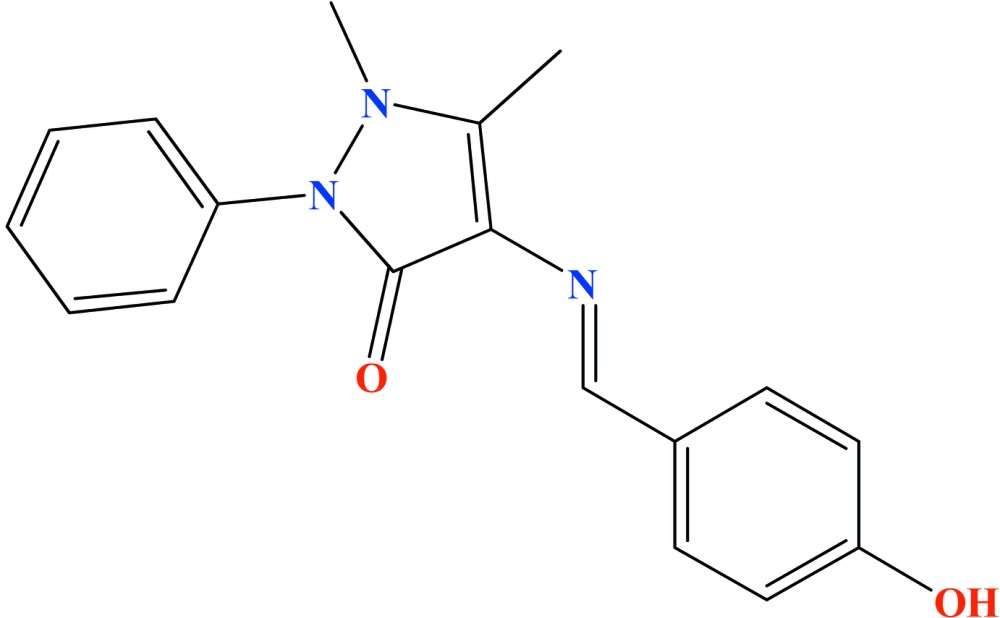


## Experimental   

### Crystal data  

C_18_H_17_N_3_O_2_
*M* *_r_* = 307.35
Triclinic, 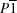

*a* = 8.1214 (7) Å
*b* = 12.5405 (10) Å
*c* = 23.1875 (19) Å
α = 91.121 (1)°
β = 90.199 (1)°
γ = 90.748 (1)°
*V* = 2360.9 (3) Å^3^
*Z* = 6
Mo *K*α radiation
μ = 0.09 mm^−1^
*T* = 150 K
0.30 × 0.18 × 0.15 mm

### Data collection  

Bruker SMART APEX CCD diffractometer
Absorption correction: multi-scan (*SADABS*; Bruker, 2015 ▸) *T*_min_ = 0.86, *T*_max_ = 0.99
22491 measured reflections
11415 independent reflections
7110 reflections with *I* > 2σ(*I*)
*R*_int_ = 0.035

### Refinement  

*R*[*F* ^2^ > 2σ(*F* ^2^)] = 0.046
*wR*(*F* ^2^) = 0.115
*S* = 0.97
11415 reflections
628 parameters
H-atom parameters constrained
Δρ_max_ = 0.25 e Å^−3^
Δρ_min_ = −0.21 e Å^−3^

### 

Data collection: *APEX2* (Bruker, 2015 ▸); cell refinement: *SAINT* (Bruker, 2015 ▸); data reduction: *SAINT*; program(s) used to solve structure: *SHELXT* (Sheldrick, 2015*b* ▸); program(s) used to refine structure: *SHELXL2014* (Sheldrick, 2015*a* ▸); molecular graphics: *DIAMOND* (Brandenburg & Putz, 2012 ▸); software used to prepare material for publication: *SHELXTL* (Sheldrick, 2008 ▸).

## Supplementary Material

Crystal structure: contains datablock(s) global, I. DOI: 10.1107/S2056989015021325/hb7540sup1.cif

Structure factors: contains datablock(s) I. DOI: 10.1107/S2056989015021325/hb7540Isup2.hkl

Supporting information file. DOI: 10.1107/S2056989015021325/hb7540Isup3.cml

**Fig. 1.**
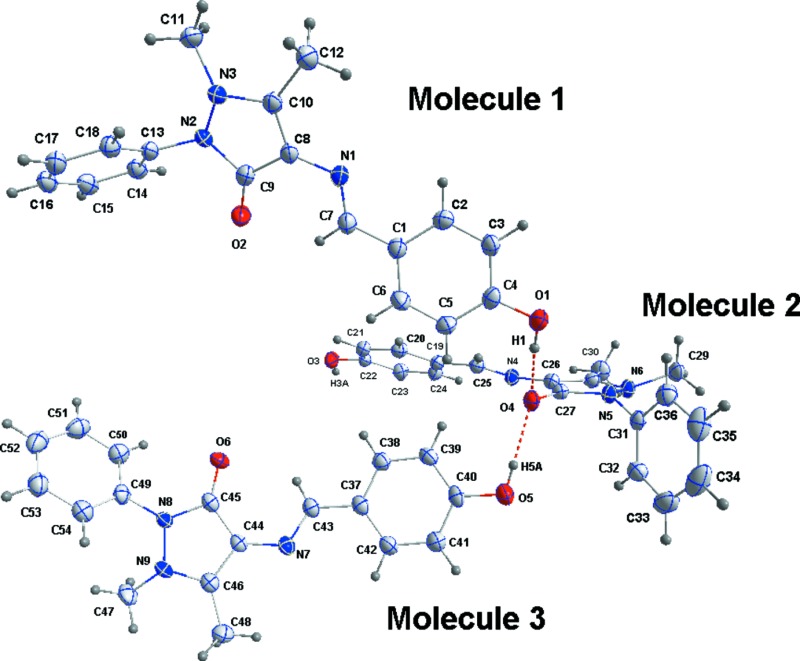
The asymmetric unit with labeling scheme and 50% probability ellipsoids. The O—H···O hydrogen bonds are shown as dotted lines.

**Fig. 2.**
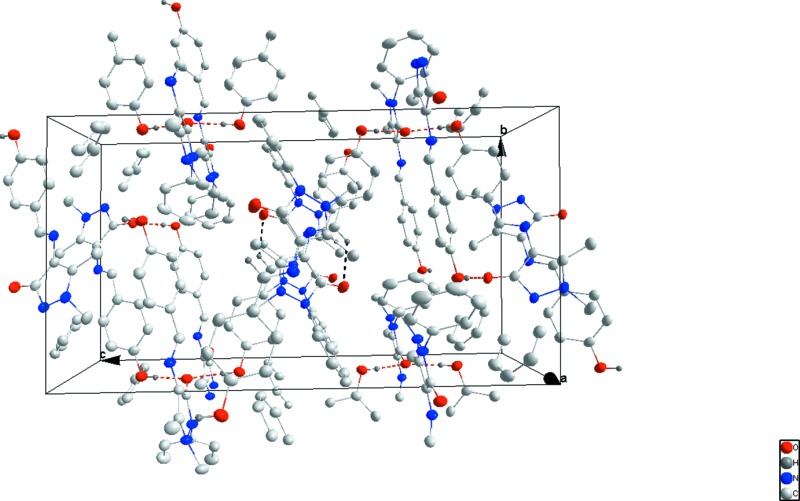
The unit-cell contents viewed down the a axis. Intermolecular O—H···O and C—H···O hydrogen bonds are shown, respectively, as red and black dotted lines.

. DOI: 10.1107/S2056989015021325/hb7540fig1.tif
The asymmetric unit with labeling scheme and 50% probability ellipsoids. The O—H⋯O hydrogen bonds are shown as dotted lines.

a . DOI: 10.1107/S2056989015021325/hb7540fig2.tif
The unit-cell contents viewed down the *a* axis. Inter­molecular O—H⋯O and C—H⋯O hydrogen bonds are shown, respectively, as red and black dotted lines.

CCDC reference: 1436039

Additional supporting information:  crystallographic information; 3D view; checkCIF report

## Figures and Tables

**Table 1:** Hydrogen-bond geometry (Å, °)

*D*—H⋯*A*	*D*—H	H⋯*A*	*D*⋯*A*	*D*—H⋯*A*
O1—H1⋯O4	0.84	1.86	2.6947 (14)	170
O3—H3*A*⋯O6^i^	0.84	1.86	2.6993 (15)	175
O5—H5*A*⋯O4	0.84	1.88	2.7218 (15)	179
C7—H7⋯O2	0.95	2.30	3.009 (2)	131
C25—H25⋯O4	0.95	2.46	3.0612 (18)	121
C43—H43⋯O6	0.95	2.36	2.9989 (18)	124
C33—H33⋯O6^ii^	0.95	2.42	3.168 (2)	135
C35—H35⋯O2^ii^	0.95	2.31	3.122 (2)	143

Symmetry codes: (i) 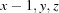; (ii) 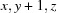.

## supplementary crystallographic information

### S1. Comment

The pyrazolone ring system is an important structural moiety found in numerous pharmaceutically active compounds. This is mainly due to the ease preparation and the important versatile pharmaceutical and industrial applications. Pyrazolones are mostly useful as anti-inflammatory and analgesic activities (Nirali & Maulik, 2010), but in recent times, they are known to exhibit anti-cancer (Rahat *et al.*, 2008), anti-bacterial (Thakkar & Joshi, 2010) and several other pharmacological actions like anti-fungal (Mahmoud *et al.*, 2011), protein kinase inhibitor (Tripathy *et al.*, 2007), anti-pyretic (Brune, 1997), anti-convulsant (Abdel-Aziz *et al.*, 2009). In addition, they have been used as plant growth regulator, herbicidal and as an azo-dyes (Karci & Ertan, 2002; Khalil *et al.*, 2005; Ho, 2005). Based on such findings and following to our on-going study, we report herein the synthesis and crystal structure of the title compound.

For the title compound there are three independent molecules in the asymmetric unit (Fig. 1) which differ in their conformations. In molecules 1 - 3, respectively, the maximum deviations from the mean planes of the pyrazolone rings are 0.0501 (8) Å (N3), 0.0305 (8) Å (N5) and -0.0367 (8) Å (N8). In molecule 1 the dihedral angles between the mean plane of the pyrazolone ring and those of the phenyl and hydroxyphenyl substituents are, respectively, 54.43 (6)° and 28.72 (6)°. In molecule 2 the corresponding angles are 86.84 (6) and 25.69 (5)° while in molecule 3 they are 47.41 (7) and 17.09 (7)°. In the asymmetric unit, molecules 1 and 3,respectively, form O1—H1···O4 and O5—H5A···O4 hydrogen bonds which tie the unit together (Fig. 1 and Table 1) while O3—H3A···O6^i^ (i: *x* - 1, *y*, *z*) hydrogen bonds aid in the packing (Fig. 2 and Table 1).

### S2. Refinement

H-atoms attached to carbon were placed in calculated positions (C—H = 0.95 - 0.98 Å) while those attached to oxygen were placed in ocations derived from a difference map and their coordinates adjusted to give O—H = 0.84 Å. All were included as riding contributions with isotropic displacement parameters 1.2 - 1.5 times those of the attached atoms.

### Crystal data

**Table:**

C_18_H_17_N_3_O_2_	*Z* = 6
*M**_r_* = 307.35	*F*(000) = 972
Triclinic, *P*1	*D*_x_ = 1.297 Mg m^−^^3^
*a* = 8.1214 (7) Å	Mo *K*α radiation, λ = 0.71073 Å
*b* = 12.5405 (10) Å	Cell parameters from 6224 reflections
*c* = 23.1875 (19) Å	θ = 2.4–28.2°
α = 91.121 (1)°	µ = 0.09 mm^−^^1^
β = 90.199 (1)°	*T* = 150 K
γ = 90.748 (1)°	Column, colourless
*V* = 2360.9 (3) Å^3^	0.30 × 0.18 × 0.15 mm

### Data collection

**Table:**

Bruker SMART APEX CCD diffractometer	11415 independent reflections
Radiation source: fine-focus sealed tube	7110 reflections with *I* > 2σ(*I*)
Graphite monochromator	*R*_int_ = 0.035
Detector resolution: 8.3333 pixels mm^-1^	θ_max_ = 28.5°, θ_min_ = 1.6°
φ and ω scans	*h* = −10→10
Absorption correction: multi-scan (*SADABS*; Bruker, 2015)	*k* = −16→16
*T*_min_ = 0.86, *T*_max_ = 0.99	*l* = −30→31
22491 measured reflections	

### Refinement

**Table:**

Refinement on *F*^2^	Primary atom site location: structure-invariant direct methods
Least-squares matrix: full	Secondary atom site location: difference Fourier map
*R*[*F*^2^ > 2σ(*F*^2^)] = 0.046	Hydrogen site location: mixed
*wR*(*F*^2^) = 0.115	H-atom parameters constrained
*S* = 0.97	*w* = 1/[σ^2^(*F*_o_^2^) + (0.0468*P*)^2^] where *P* = (*F*_o_^2^ + 2*F*_c_^2^)/3
11415 reflections	(Δ/σ)_max_ = 0.001
628 parameters	Δρ_max_ = 0.25 e Å^−^^3^
0 restraints	Δρ_min_ = −0.21 e Å^−^^3^

### Special details

**Table:**

Experimental. The diffraction data were collected in three sets of 363 frames (0.5° width in ω) at φ = 0, 120 and 240°. A scan time of 40 sec/frame was used.
Geometry. All e.s.d.'s (except the e.s.d. in the dihedral angle between two l.s. planes) are estimated using the full covariance matrix. The cell e.s.d.'s are taken into account individually in the estimation of e.s.d.'s in distances, angles and torsion angles; correlations between e.s.d.'s in cell parameters are only used when they are defined by crystal symmetry. An approximate (isotropic) treatment of cell e.s.d.'s is used for estimating e.s.d.'s involving l.s. planes.
Refinement. Refinement of *F*^2^ against ALL reflections. The weighted *R*-factor *wR* and goodness of fit *S* are based on *F*^2^, conventional *R*-factors *R* are based on *F*, with *F* set to zero for negative *F*^2^. The threshold expression of *F*^2^ > σ(*F*^2^) is used only for calculating *R*-factors(gt) *etc*. and is not relevant to the choice of reflections for refinement. *R*-factors based on *F*^2^ are statistically about twice as large as those based on *F*, and *R*- factors based on ALL data will be even larger. H-atoms attached to carbon were placed in calculated positions (C—H = 0.95 - 0.98 Å) while those attached to oxygen were placed in ocations derived from a difference map and their coordinates adjusted to give O—H = 0.84%A. All were included as riding contributions with isotropic displacement parameters 1.2 - 1.5 times those of the attached atoms.

### Fractional atomic coordinates and isotropic or equivalent isotropic displacement parameters (Å^2^)

**Table:**

	*x*	*y*	*z*	*U*_iso_*/*U*_eq_	
O1	0.43335 (13)	0.99066 (8)	0.36405 (5)	0.0329 (3)	
H1	0.4041	0.9909	0.3293	0.040*	
O2	0.69023 (15)	0.34940 (9)	0.41279 (5)	0.0414 (3)	
N1	0.69265 (15)	0.56857 (10)	0.48144 (5)	0.0282 (3)	
N2	0.82894 (15)	0.29591 (10)	0.49432 (5)	0.0264 (3)	
N3	0.89959 (15)	0.34786 (10)	0.54289 (5)	0.0261 (3)	
C1	0.57962 (19)	0.68917 (12)	0.41316 (7)	0.0300 (4)	
C2	0.54167 (18)	0.76897 (12)	0.45379 (7)	0.0282 (4)	
H2	0.5501	0.7548	0.4938	0.034*	
C3	0.49214 (18)	0.86797 (12)	0.43633 (7)	0.0287 (4)	
H3	0.4660	0.9213	0.4644	0.034*	
C4	0.48012 (18)	0.89043 (12)	0.37808 (7)	0.0268 (3)	
C5	0.5166 (2)	0.81240 (13)	0.33727 (7)	0.0338 (4)	
H5	0.5087	0.8271	0.2973	0.041*	
C6	0.5647 (2)	0.71295 (13)	0.35493 (7)	0.0386 (4)	
H6	0.5881	0.6594	0.3267	0.046*	
C7	0.6392 (2)	0.58501 (13)	0.43047 (7)	0.0338 (4)	
H7	0.6381	0.5278	0.4030	0.041*	
C8	0.75333 (18)	0.46864 (12)	0.49509 (6)	0.0259 (3)	
C9	0.74727 (19)	0.37078 (12)	0.46124 (7)	0.0290 (4)	
C10	0.84009 (18)	0.44934 (12)	0.54448 (6)	0.0260 (3)	
C11	0.9202 (2)	0.28398 (13)	0.59429 (6)	0.0331 (4)	
H11A	0.9757	0.3270	0.6245	0.050*	
H11B	0.9867	0.2214	0.5848	0.050*	
H11C	0.8119	0.2606	0.6081	0.050*	
C12	0.8719 (2)	0.52260 (13)	0.59430 (7)	0.0379 (4)	
H12A	0.8126	0.4967	0.6281	0.057*	
H12B	0.8340	0.5942	0.5851	0.057*	
H12C	0.9903	0.5253	0.6027	0.057*	
C13	0.90702 (19)	0.20424 (11)	0.47004 (6)	0.0253 (3)	
C14	0.80958 (19)	0.12678 (12)	0.44266 (6)	0.0284 (4)	
H14	0.6932	0.1332	0.4419	0.034*	
C15	0.8844 (2)	0.03966 (12)	0.41634 (7)	0.0318 (4)	
H15	0.8190	−0.0136	0.3972	0.038*	
C16	1.0533 (2)	0.03015 (12)	0.41783 (7)	0.0319 (4)	
H16	1.1039	−0.0295	0.3997	0.038*	
C17	1.1490 (2)	0.10741 (12)	0.44578 (7)	0.0321 (4)	
H17	1.2652	0.0999	0.4472	0.038*	
C18	1.07674 (19)	0.19580 (12)	0.47182 (6)	0.0277 (3)	
H18	1.1426	0.2494	0.4905	0.033*	
O3	−0.07276 (14)	0.38608 (8)	0.19190 (5)	0.0335 (3)	
H3A	−0.1490	0.3819	0.1674	0.040*	
O4	0.30090 (12)	0.99610 (8)	0.25745 (4)	0.0276 (2)	
N4	−0.05124 (15)	0.88981 (10)	0.24542 (5)	0.0269 (3)	
N5	0.14278 (14)	1.14370 (9)	0.27759 (5)	0.0238 (3)	
N6	−0.02363 (14)	1.16934 (9)	0.27561 (5)	0.0241 (3)	
C19	0.00875 (18)	0.70312 (11)	0.24138 (6)	0.0250 (3)	
C20	0.12014 (19)	0.62286 (12)	0.25460 (6)	0.0272 (3)	
H20	0.2185	0.6414	0.2749	0.033*	
C21	0.09102 (19)	0.51762 (12)	0.23895 (6)	0.0274 (3)	
H21	0.1682	0.4645	0.2487	0.033*	
C22	−0.05170 (19)	0.48969 (11)	0.20883 (6)	0.0254 (3)	
C23	−0.16547 (19)	0.56840 (12)	0.19590 (6)	0.0282 (4)	
H23	−0.2644	0.5496	0.1760	0.034*	
C24	−0.13492 (19)	0.67315 (12)	0.21193 (6)	0.0278 (3)	
H24	−0.2132	0.7259	0.2027	0.033*	
C25	0.04643 (19)	0.81384 (12)	0.25703 (6)	0.0267 (3)	
H25	0.1470	0.8302	0.2765	0.032*	
C26	−0.00185 (18)	0.99402 (11)	0.25961 (6)	0.0234 (3)	
C27	0.15973 (18)	1.03778 (11)	0.26465 (6)	0.0231 (3)	
C28	−0.10963 (18)	1.07782 (12)	0.26662 (6)	0.0250 (3)	
C29	−0.07842 (19)	1.26236 (11)	0.30874 (7)	0.0297 (4)	
H29A	−0.1949	1.2749	0.3005	0.045*	
H29B	−0.0129	1.3250	0.2980	0.045*	
H29C	−0.0642	1.2496	0.3500	0.045*	
C30	−0.29252 (18)	1.07551 (13)	0.26618 (8)	0.0356 (4)	
H30A	−0.3332	1.0926	0.3050	0.053*	
H30B	−0.3320	1.0043	0.2542	0.053*	
H30C	−0.3330	1.1282	0.2391	0.053*	
C31	0.26579 (17)	1.22415 (11)	0.26718 (6)	0.0225 (3)	
C32	0.3001 (2)	1.25156 (12)	0.21120 (7)	0.0318 (4)	
H32	0.2406	1.2189	0.1801	0.038*	
C33	0.4207 (2)	1.32638 (14)	0.20060 (8)	0.0422 (4)	
H33	0.4461	1.3451	0.1621	0.051*	
C34	0.5045 (2)	1.37399 (14)	0.24610 (9)	0.0456 (5)	
H34	0.5874	1.4261	0.2388	0.055*	
C35	0.4699 (2)	1.34720 (14)	0.30215 (9)	0.0435 (5)	
H35	0.5280	1.3811	0.3332	0.052*	
C36	0.3501 (2)	1.27070 (12)	0.31313 (7)	0.0321 (4)	
H36	0.3264	1.2507	0.3515	0.039*	
O5	0.43831 (13)	1.00160 (8)	0.15063 (5)	0.0359 (3)	
H5A	0.3963	1.0005	0.1837	0.043*	
O6	0.69633 (13)	0.37408 (8)	0.10793 (4)	0.0302 (3)	
N7	0.68224 (15)	0.57227 (10)	0.02772 (5)	0.0278 (3)	
N8	0.82733 (15)	0.30098 (10)	0.02799 (5)	0.0255 (3)	
N9	0.88764 (15)	0.34003 (10)	−0.02407 (5)	0.0277 (3)	
C37	0.53272 (18)	0.69523 (12)	0.08808 (7)	0.0277 (4)	
C38	0.46494 (18)	0.71054 (13)	0.14274 (7)	0.0297 (4)	
H38	0.4420	0.6503	0.1657	0.036*	
C39	0.43024 (18)	0.81153 (12)	0.16433 (7)	0.0286 (4)	
H39	0.3844	0.8201	0.2017	0.034*	
C40	0.46269 (18)	0.89989 (12)	0.13116 (7)	0.0279 (4)	
C41	0.5231 (2)	0.88589 (13)	0.07559 (7)	0.0347 (4)	
H41	0.5407	0.9461	0.0520	0.042*	
C42	0.55773 (19)	0.78528 (13)	0.05449 (7)	0.0326 (4)	
H42	0.5993	0.7770	0.0164	0.039*	
C43	0.58390 (18)	0.58873 (12)	0.07025 (7)	0.0278 (4)	
H43	0.5431	0.5292	0.0908	0.033*	
C44	0.74040 (17)	0.46900 (12)	0.01784 (6)	0.0242 (3)	
C45	0.74410 (17)	0.38185 (12)	0.05700 (6)	0.0244 (3)	
C46	0.82686 (18)	0.43964 (12)	−0.03047 (6)	0.0253 (3)	
C47	0.9212 (2)	0.26398 (13)	−0.07089 (7)	0.0368 (4)	
H47A	0.8227	0.2200	−0.0786	0.055*	
H47B	1.0124	0.2181	−0.0598	0.055*	
H47C	0.9512	0.3028	−0.1057	0.055*	
C48	0.8547 (2)	0.50055 (13)	−0.08359 (7)	0.0344 (4)	
H48A	0.9725	0.5017	−0.0926	0.052*	
H48B	0.8165	0.5738	−0.0777	0.052*	
H48C	0.7935	0.4666	−0.1156	0.052*	
C49	0.90208 (19)	0.21186 (12)	0.05461 (6)	0.0275 (3)	
C50	0.8033 (2)	0.14313 (13)	0.08618 (7)	0.0346 (4)	
H50	0.6883	0.1546	0.0893	0.041*	
C51	0.8750 (2)	0.05781 (14)	0.11303 (7)	0.0413 (4)	
H51	0.8091	0.0112	0.1354	0.050*	
C52	1.0412 (2)	0.03980 (14)	0.10757 (7)	0.0413 (4)	
H52	1.0892	−0.0196	0.1257	0.050*	
C53	1.1383 (2)	0.10819 (14)	0.07575 (7)	0.0389 (4)	
H53	1.2528	0.0952	0.0718	0.047*	
C54	1.06954 (19)	0.19547 (13)	0.04960 (7)	0.0328 (4)	
H54	1.1365	0.2435	0.0285	0.039*	

### Atomic displacement parameters (Å^2^)

**Table:**

	*U*^11^	*U*^22^	*U*^33^	*U*^12^	*U*^13^	*U*^23^
O1	0.0364 (6)	0.0284 (6)	0.0344 (6)	0.0082 (5)	0.0011 (5)	0.0044 (5)
O2	0.0553 (8)	0.0376 (7)	0.0314 (7)	0.0158 (6)	−0.0136 (6)	−0.0071 (5)
N1	0.0293 (7)	0.0247 (7)	0.0308 (7)	0.0052 (6)	0.0009 (6)	0.0013 (6)
N2	0.0314 (7)	0.0248 (7)	0.0229 (7)	0.0054 (6)	−0.0012 (5)	−0.0035 (5)
N3	0.0327 (7)	0.0242 (7)	0.0214 (7)	0.0034 (6)	−0.0009 (5)	−0.0002 (5)
C1	0.0314 (9)	0.0266 (9)	0.0321 (9)	0.0043 (7)	−0.0041 (7)	−0.0005 (7)
C2	0.0259 (8)	0.0320 (9)	0.0268 (8)	0.0008 (7)	−0.0002 (7)	0.0014 (7)
C3	0.0284 (9)	0.0276 (9)	0.0302 (9)	0.0053 (7)	0.0026 (7)	−0.0025 (7)
C4	0.0203 (8)	0.0260 (8)	0.0341 (9)	0.0025 (6)	−0.0003 (6)	0.0029 (7)
C5	0.0409 (10)	0.0354 (10)	0.0255 (9)	0.0088 (8)	−0.0027 (7)	0.0028 (7)
C6	0.0522 (11)	0.0357 (10)	0.0280 (9)	0.0123 (8)	−0.0034 (8)	−0.0060 (7)
C7	0.0406 (10)	0.0264 (9)	0.0345 (10)	0.0079 (7)	−0.0024 (8)	−0.0034 (7)
C8	0.0278 (8)	0.0226 (8)	0.0275 (8)	0.0040 (6)	0.0034 (7)	−0.0005 (6)
C9	0.0303 (9)	0.0287 (9)	0.0281 (9)	0.0069 (7)	−0.0005 (7)	0.0011 (7)
C10	0.0296 (8)	0.0220 (8)	0.0265 (8)	0.0019 (6)	0.0047 (7)	0.0013 (6)
C11	0.0453 (10)	0.0304 (9)	0.0240 (9)	0.0043 (8)	−0.0014 (7)	0.0040 (7)
C12	0.0550 (11)	0.0292 (9)	0.0296 (9)	0.0049 (8)	−0.0027 (8)	−0.0024 (7)
C13	0.0335 (9)	0.0196 (8)	0.0230 (8)	0.0044 (7)	0.0031 (6)	0.0014 (6)
C14	0.0302 (9)	0.0272 (9)	0.0279 (8)	−0.0004 (7)	0.0040 (7)	0.0012 (7)
C15	0.0417 (10)	0.0230 (9)	0.0306 (9)	−0.0041 (7)	0.0045 (7)	−0.0010 (7)
C16	0.0424 (10)	0.0234 (9)	0.0300 (9)	0.0055 (7)	0.0064 (7)	−0.0003 (7)
C17	0.0326 (9)	0.0291 (9)	0.0348 (9)	0.0080 (7)	0.0024 (7)	0.0020 (7)
C18	0.0307 (9)	0.0244 (8)	0.0279 (8)	0.0024 (7)	−0.0014 (7)	−0.0001 (7)
O3	0.0453 (7)	0.0227 (6)	0.0324 (6)	0.0005 (5)	−0.0041 (5)	−0.0029 (5)
O4	0.0228 (6)	0.0250 (6)	0.0351 (6)	0.0051 (4)	0.0010 (5)	−0.0013 (5)
N4	0.0295 (7)	0.0217 (7)	0.0295 (7)	−0.0011 (6)	0.0007 (6)	−0.0015 (5)
N5	0.0186 (6)	0.0204 (7)	0.0323 (7)	0.0015 (5)	0.0031 (5)	−0.0004 (5)
N6	0.0193 (6)	0.0205 (7)	0.0326 (7)	0.0030 (5)	0.0035 (5)	−0.0005 (5)
C19	0.0300 (8)	0.0215 (8)	0.0237 (8)	−0.0008 (6)	0.0032 (6)	0.0003 (6)
C20	0.0286 (8)	0.0281 (9)	0.0249 (8)	−0.0008 (7)	−0.0001 (6)	0.0007 (6)
C21	0.0332 (9)	0.0236 (8)	0.0256 (8)	0.0051 (7)	0.0017 (7)	0.0023 (6)
C22	0.0356 (9)	0.0185 (8)	0.0222 (8)	−0.0012 (7)	0.0049 (7)	0.0005 (6)
C23	0.0282 (8)	0.0281 (9)	0.0281 (9)	−0.0022 (7)	−0.0012 (7)	0.0006 (7)
C24	0.0289 (9)	0.0236 (8)	0.0311 (9)	0.0024 (7)	0.0005 (7)	0.0032 (7)
C25	0.0288 (8)	0.0252 (9)	0.0260 (8)	−0.0020 (7)	0.0005 (7)	0.0002 (6)
C26	0.0252 (8)	0.0210 (8)	0.0241 (8)	0.0004 (6)	0.0008 (6)	0.0007 (6)
C27	0.0261 (8)	0.0221 (8)	0.0213 (8)	0.0022 (6)	0.0021 (6)	0.0016 (6)
C28	0.0252 (8)	0.0250 (8)	0.0249 (8)	−0.0006 (6)	0.0015 (6)	0.0022 (6)
C29	0.0302 (9)	0.0221 (8)	0.0371 (9)	0.0067 (7)	0.0084 (7)	0.0003 (7)
C30	0.0233 (8)	0.0358 (10)	0.0476 (11)	−0.0005 (7)	0.0032 (7)	−0.0003 (8)
C31	0.0185 (7)	0.0188 (8)	0.0304 (8)	0.0024 (6)	0.0020 (6)	−0.0004 (6)
C32	0.0338 (9)	0.0308 (9)	0.0309 (9)	0.0009 (7)	0.0038 (7)	−0.0024 (7)
C33	0.0394 (10)	0.0380 (10)	0.0495 (11)	0.0006 (8)	0.0162 (9)	0.0093 (9)
C34	0.0252 (9)	0.0309 (10)	0.0810 (15)	−0.0023 (8)	0.0056 (9)	0.0093 (10)
C35	0.0346 (10)	0.0322 (10)	0.0633 (13)	−0.0007 (8)	−0.0179 (9)	−0.0042 (9)
C36	0.0352 (9)	0.0287 (9)	0.0323 (9)	0.0033 (7)	−0.0067 (7)	−0.0003 (7)
O5	0.0404 (7)	0.0316 (7)	0.0357 (7)	0.0082 (5)	0.0042 (5)	−0.0045 (5)
O6	0.0321 (6)	0.0348 (6)	0.0237 (6)	0.0006 (5)	0.0063 (5)	−0.0028 (5)
N7	0.0279 (7)	0.0304 (7)	0.0250 (7)	0.0035 (6)	−0.0018 (6)	−0.0042 (6)
N8	0.0264 (7)	0.0277 (7)	0.0223 (7)	0.0023 (6)	0.0043 (5)	−0.0024 (5)
N9	0.0285 (7)	0.0320 (8)	0.0225 (7)	0.0011 (6)	0.0050 (5)	−0.0053 (6)
C37	0.0209 (8)	0.0321 (9)	0.0299 (9)	0.0032 (7)	−0.0005 (6)	−0.0026 (7)
C38	0.0276 (9)	0.0322 (9)	0.0293 (9)	0.0029 (7)	0.0016 (7)	0.0004 (7)
C39	0.0257 (8)	0.0353 (9)	0.0247 (8)	0.0049 (7)	0.0013 (6)	−0.0028 (7)
C40	0.0228 (8)	0.0286 (9)	0.0323 (9)	0.0068 (7)	−0.0020 (7)	−0.0044 (7)
C41	0.0377 (10)	0.0328 (10)	0.0338 (9)	0.0046 (7)	0.0047 (7)	0.0026 (7)
C42	0.0311 (9)	0.0364 (10)	0.0303 (9)	0.0035 (7)	0.0063 (7)	−0.0006 (7)
C43	0.0229 (8)	0.0298 (9)	0.0306 (9)	0.0013 (7)	−0.0005 (7)	−0.0026 (7)
C44	0.0219 (8)	0.0266 (8)	0.0238 (8)	−0.0006 (6)	0.0000 (6)	−0.0039 (6)
C45	0.0178 (7)	0.0290 (9)	0.0262 (8)	−0.0009 (6)	−0.0002 (6)	−0.0058 (7)
C46	0.0212 (8)	0.0294 (9)	0.0251 (8)	−0.0011 (6)	−0.0022 (6)	−0.0039 (6)
C47	0.0420 (10)	0.0414 (10)	0.0268 (9)	0.0097 (8)	0.0018 (7)	−0.0101 (7)
C48	0.0369 (10)	0.0386 (10)	0.0276 (9)	−0.0012 (8)	0.0040 (7)	−0.0008 (7)
C49	0.0303 (9)	0.0244 (8)	0.0276 (8)	0.0025 (7)	−0.0006 (7)	−0.0054 (6)
C50	0.0330 (9)	0.0317 (9)	0.0388 (10)	−0.0027 (7)	0.0010 (7)	−0.0014 (8)
C51	0.0495 (12)	0.0335 (10)	0.0408 (11)	−0.0041 (8)	−0.0002 (9)	0.0038 (8)
C52	0.0519 (12)	0.0343 (10)	0.0378 (10)	0.0088 (9)	−0.0068 (9)	−0.0013 (8)
C53	0.0340 (10)	0.0434 (11)	0.0394 (10)	0.0113 (8)	−0.0032 (8)	−0.0013 (8)
C54	0.0296 (9)	0.0351 (10)	0.0335 (9)	0.0013 (7)	0.0019 (7)	−0.0040 (7)

### Geometric parameters (Å, º)

**Table:**

O1—C4	1.3630 (17)	C25—H25	0.9500
O1—H1	0.8402	C26—C28	1.3839 (19)
O2—C9	1.2369 (18)	C26—C27	1.419 (2)
N1—C7	1.2791 (19)	C28—C30	1.485 (2)
N1—C8	1.3942 (18)	C29—H29A	0.9800
N2—C9	1.3966 (18)	C29—H29B	0.9800
N2—N3	1.4069 (16)	C29—H29C	0.9800
N2—C13	1.4272 (18)	C30—H30A	0.9800
N3—C10	1.3669 (18)	C30—H30B	0.9800
N3—C11	1.4593 (18)	C30—H30C	0.9800
C1—C6	1.394 (2)	C31—C32	1.378 (2)
C1—C2	1.399 (2)	C31—C36	1.380 (2)
C1—C7	1.462 (2)	C32—C33	1.374 (2)
C2—C3	1.378 (2)	C32—H32	0.9500
C2—H2	0.9500	C33—C34	1.376 (3)
C3—C4	1.388 (2)	C33—H33	0.9500
C3—H3	0.9500	C34—C35	1.378 (3)
C4—C5	1.383 (2)	C34—H34	0.9500
C5—C6	1.381 (2)	C35—C36	1.385 (2)
C5—H5	0.9500	C35—H35	0.9500
C6—H6	0.9500	C36—H36	0.9500
C7—H7	0.9500	O5—C40	1.3617 (17)
C8—C10	1.370 (2)	O5—H5A	0.8402
C8—C9	1.444 (2)	O6—C45	1.2496 (17)
C10—C12	1.482 (2)	N7—C43	1.2863 (18)
C11—H11A	0.9800	N7—C44	1.3992 (18)
C11—H11B	0.9800	N8—C45	1.3900 (18)
C11—H11C	0.9800	N8—N9	1.3993 (16)
C12—H12A	0.9800	N8—C49	1.4286 (19)
C12—H12B	0.9800	N9—C46	1.3597 (18)
C12—H12C	0.9800	N9—C47	1.4597 (18)
C13—C18	1.384 (2)	C37—C38	1.395 (2)
C13—C14	1.388 (2)	C37—C42	1.397 (2)
C14—C15	1.389 (2)	C37—C43	1.457 (2)
C14—H14	0.9500	C38—C39	1.385 (2)
C15—C16	1.378 (2)	C38—H38	0.9500
C15—H15	0.9500	C39—C40	1.384 (2)
C16—C17	1.384 (2)	C39—H39	0.9500
C16—H16	0.9500	C40—C41	1.389 (2)
C17—C18	1.390 (2)	C41—C42	1.377 (2)
C17—H17	0.9500	C41—H41	0.9500
C18—H18	0.9500	C42—H42	0.9500
O3—C22	1.3584 (17)	C43—H43	0.9500
O3—H3A	0.8402	C44—C46	1.371 (2)
O4—C27	1.2763 (16)	C44—C45	1.435 (2)
N4—C25	1.2798 (18)	C46—C48	1.479 (2)
N4—C26	1.3949 (18)	C47—H47A	0.9800
N5—C27	1.3648 (18)	C47—H47B	0.9800
N5—N6	1.3941 (16)	C47—H47C	0.9800
N5—C31	1.4350 (18)	C48—H48A	0.9800
N6—C28	1.3482 (18)	C48—H48B	0.9800
N6—C29	1.4593 (17)	C48—H48C	0.9800
C19—C24	1.394 (2)	C49—C54	1.383 (2)
C19—C20	1.400 (2)	C49—C50	1.390 (2)
C19—C25	1.457 (2)	C50—C51	1.382 (2)
C20—C21	1.379 (2)	C50—H50	0.9500
C20—H20	0.9500	C51—C52	1.378 (2)
C21—C22	1.389 (2)	C51—H51	0.9500
C21—H21	0.9500	C52—C53	1.383 (2)
C22—C23	1.397 (2)	C52—H52	0.9500
C23—C24	1.377 (2)	C53—C54	1.385 (2)
C23—H23	0.9500	C53—H53	0.9500
C24—H24	0.9500	C54—H54	0.9500
			
C4—O1—H1	109.3	N5—C27—C26	106.62 (12)
C7—N1—C8	119.66 (13)	N6—C28—C26	109.55 (13)
C9—N2—N3	109.04 (12)	N6—C28—C30	121.58 (13)
C9—N2—C13	123.24 (12)	C26—C28—C30	128.85 (14)
N3—N2—C13	119.37 (12)	N6—C29—H29A	109.5
C10—N3—N2	106.96 (11)	N6—C29—H29B	109.5
C10—N3—C11	123.17 (12)	H29A—C29—H29B	109.5
N2—N3—C11	116.63 (12)	N6—C29—H29C	109.5
C6—C1—C2	117.94 (14)	H29A—C29—H29C	109.5
C6—C1—C7	120.32 (15)	H29B—C29—H29C	109.5
C2—C1—C7	121.70 (14)	C28—C30—H30A	109.5
C3—C2—C1	120.59 (14)	C28—C30—H30B	109.5
C3—C2—H2	119.7	H30A—C30—H30B	109.5
C1—C2—H2	119.7	C28—C30—H30C	109.5
C2—C3—C4	120.53 (14)	H30A—C30—H30C	109.5
C2—C3—H3	119.7	H30B—C30—H30C	109.5
C4—C3—H3	119.7	C32—C31—C36	121.27 (14)
O1—C4—C5	123.04 (14)	C32—C31—N5	119.12 (13)
O1—C4—C3	117.26 (13)	C36—C31—N5	119.59 (14)
C5—C4—C3	119.70 (14)	C33—C32—C31	119.66 (16)
C6—C5—C4	119.61 (15)	C33—C32—H32	120.2
C6—C5—H5	120.2	C31—C32—H32	120.2
C4—C5—H5	120.2	C32—C33—C34	119.56 (17)
C5—C6—C1	121.62 (15)	C32—C33—H33	120.2
C5—C6—H6	119.2	C34—C33—H33	120.2
C1—C6—H6	119.2	C33—C34—C35	120.93 (16)
N1—C7—C1	122.03 (15)	C33—C34—H34	119.5
N1—C7—H7	119.0	C35—C34—H34	119.5
C1—C7—H7	119.0	C34—C35—C36	119.84 (16)
C10—C8—N1	123.46 (14)	C34—C35—H35	120.1
C10—C8—C9	107.64 (13)	C36—C35—H35	120.1
N1—C8—C9	128.82 (13)	C31—C36—C35	118.73 (16)
O2—C9—N2	122.70 (14)	C31—C36—H36	120.6
O2—C9—C8	131.93 (14)	C35—C36—H36	120.6
N2—C9—C8	105.34 (13)	C40—O5—H5A	109.6
N3—C10—C8	110.19 (13)	C43—N7—C44	118.60 (13)
N3—C10—C12	121.57 (13)	C45—N8—N9	109.06 (12)
C8—C10—C12	128.24 (14)	C45—N8—C49	125.18 (12)
N3—C11—H11A	109.5	N9—N8—C49	120.77 (11)
N3—C11—H11B	109.5	C46—N9—N8	107.53 (11)
H11A—C11—H11B	109.5	C46—N9—C47	125.54 (13)
N3—C11—H11C	109.5	N8—N9—C47	118.42 (12)
H11A—C11—H11C	109.5	C38—C37—C42	117.58 (14)
H11B—C11—H11C	109.5	C38—C37—C43	119.10 (14)
C10—C12—H12A	109.5	C42—C37—C43	123.19 (14)
C10—C12—H12B	109.5	C39—C38—C37	121.56 (15)
H12A—C12—H12B	109.5	C39—C38—H38	119.2
C10—C12—H12C	109.5	C37—C38—H38	119.2
H12A—C12—H12C	109.5	C40—C39—C38	119.73 (14)
H12B—C12—H12C	109.5	C40—C39—H39	120.1
C18—C13—C14	121.21 (14)	C38—C39—H39	120.1
C18—C13—N2	120.28 (14)	O5—C40—C39	122.79 (14)
C14—C13—N2	118.45 (13)	O5—C40—C41	117.71 (14)
C13—C14—C15	119.14 (14)	C39—C40—C41	119.50 (14)
C13—C14—H14	120.4	C42—C41—C40	120.38 (15)
C15—C14—H14	120.4	C42—C41—H41	119.8
C16—C15—C14	120.28 (15)	C40—C41—H41	119.8
C16—C15—H15	119.9	C41—C42—C37	121.12 (15)
C14—C15—H15	119.9	C41—C42—H42	119.4
C15—C16—C17	120.02 (15)	C37—C42—H42	119.4
C15—C16—H16	120.0	N7—C43—C37	122.46 (15)
C17—C16—H16	120.0	N7—C43—H43	118.8
C16—C17—C18	120.62 (15)	C37—C43—H43	118.8
C16—C17—H17	119.7	C46—C44—N7	123.26 (14)
C18—C17—H17	119.7	C46—C44—C45	107.84 (13)
C13—C18—C17	118.72 (15)	N7—C44—C45	128.33 (13)
C13—C18—H18	120.6	O6—C45—N8	122.91 (14)
C17—C18—H18	120.6	O6—C45—C44	131.57 (14)
C22—O3—H3A	109.0	N8—C45—C44	105.39 (13)
C25—N4—C26	118.39 (13)	N9—C46—C44	109.75 (13)
C27—N5—N6	109.15 (11)	N9—C46—C48	121.71 (13)
C27—N5—C31	124.66 (12)	C44—C46—C48	128.54 (14)
N6—N5—C31	119.98 (11)	N9—C47—H47A	109.5
C28—N6—N5	107.53 (11)	N9—C47—H47B	109.5
C28—N6—C29	126.16 (12)	H47A—C47—H47B	109.5
N5—N6—C29	118.27 (12)	N9—C47—H47C	109.5
C24—C19—C20	117.65 (14)	H47A—C47—H47C	109.5
C24—C19—C25	122.16 (14)	H47B—C47—H47C	109.5
C20—C19—C25	120.16 (14)	C46—C48—H48A	109.5
C21—C20—C19	121.82 (15)	C46—C48—H48B	109.5
C21—C20—H20	119.1	H48A—C48—H48B	109.5
C19—C20—H20	119.1	C46—C48—H48C	109.5
C20—C21—C22	119.61 (14)	H48A—C48—H48C	109.5
C20—C21—H21	120.2	H48B—C48—H48C	109.5
C22—C21—H21	120.2	C54—C49—C50	120.93 (15)
O3—C22—C21	117.97 (13)	C54—C49—N8	120.48 (14)
O3—C22—C23	122.58 (14)	C50—C49—N8	118.58 (14)
C21—C22—C23	119.44 (14)	C51—C50—C49	119.05 (16)
C24—C23—C22	120.30 (14)	C51—C50—H50	120.5
C24—C23—H23	119.9	C49—C50—H50	120.5
C22—C23—H23	119.9	C52—C51—C50	120.49 (17)
C23—C24—C19	121.16 (14)	C52—C51—H51	119.8
C23—C24—H24	119.4	C50—C51—H51	119.8
C19—C24—H24	119.4	C51—C52—C53	120.06 (16)
N4—C25—C19	122.26 (14)	C51—C52—H52	120.0
N4—C25—H25	118.9	C53—C52—H52	120.0
C19—C25—H25	118.9	C52—C53—C54	120.32 (16)
C28—C26—N4	123.84 (13)	C52—C53—H53	119.8
C28—C26—C27	106.85 (13)	C54—C53—H53	119.8
N4—C26—C27	129.10 (13)	C49—C54—C53	119.14 (16)
O4—C27—N5	121.76 (13)	C49—C54—H54	120.4
O4—C27—C26	131.57 (14)	C53—C54—H54	120.4
			
C9—N2—N3—C10	9.42 (15)	N4—C26—C27—O4	0.6 (3)
C13—N2—N3—C10	158.68 (12)	C28—C26—C27—N5	3.35 (16)
C9—N2—N3—C11	151.86 (13)	N4—C26—C27—N5	178.09 (14)
C13—N2—N3—C11	−58.87 (17)	N5—N6—C28—C26	−3.20 (16)
C6—C1—C2—C3	−0.4 (2)	C29—N6—C28—C26	−150.99 (14)
C7—C1—C2—C3	177.34 (15)	N5—N6—C28—C30	175.78 (13)
C1—C2—C3—C4	−0.4 (2)	C29—N6—C28—C30	28.0 (2)
C2—C3—C4—O1	−178.79 (13)	N4—C26—C28—N6	−175.15 (13)
C2—C3—C4—C5	0.6 (2)	C27—C26—C28—N6	−0.06 (16)
O1—C4—C5—C6	179.40 (15)	N4—C26—C28—C30	6.0 (2)
C3—C4—C5—C6	0.0 (2)	C27—C26—C28—C30	−178.94 (15)
C4—C5—C6—C1	−0.9 (3)	C27—N5—C31—C32	69.87 (18)
C2—C1—C6—C5	1.1 (3)	N6—N5—C31—C32	−79.37 (17)
C7—C1—C6—C5	−176.72 (16)	C27—N5—C31—C36	−108.60 (17)
C8—N1—C7—C1	−177.79 (14)	N6—N5—C31—C36	102.17 (16)
C6—C1—C7—N1	162.55 (16)	C36—C31—C32—C33	0.2 (2)
C2—C1—C7—N1	−15.1 (3)	N5—C31—C32—C33	−178.24 (14)
C7—N1—C8—C10	167.52 (15)	C31—C32—C33—C34	−0.8 (2)
C7—N1—C8—C9	−8.9 (2)	C32—C33—C34—C35	0.4 (3)
N3—N2—C9—O2	171.47 (15)	C33—C34—C35—C36	0.5 (3)
C13—N2—C9—O2	23.6 (2)	C32—C31—C36—C35	0.7 (2)
N3—N2—C9—C8	−6.85 (16)	N5—C31—C36—C35	179.17 (13)
C13—N2—C9—C8	−154.68 (13)	C34—C35—C36—C31	−1.1 (2)
C10—C8—C9—O2	−176.28 (18)	C45—N8—N9—C46	−6.79 (15)
N1—C8—C9—O2	0.5 (3)	C49—N8—N9—C46	−163.01 (13)
C10—C8—C9—N2	1.81 (17)	C45—N8—N9—C47	−156.57 (13)
N1—C8—C9—N2	178.63 (14)	C49—N8—N9—C47	47.21 (18)
N2—N3—C10—C8	−8.28 (16)	C42—C37—C38—C39	−2.9 (2)
C11—N3—C10—C8	−147.67 (14)	C43—C37—C38—C39	173.08 (14)
N2—N3—C10—C12	172.00 (14)	C37—C38—C39—C40	0.2 (2)
C11—N3—C10—C12	32.6 (2)	C38—C39—C40—O5	−177.08 (14)
N1—C8—C10—N3	−172.97 (13)	C38—C39—C40—C41	2.8 (2)
C9—C8—C10—N3	4.06 (17)	O5—C40—C41—C42	176.88 (14)
N1—C8—C10—C12	6.7 (3)	C39—C40—C41—C42	−3.0 (2)
C9—C8—C10—C12	−176.24 (15)	C40—C41—C42—C37	0.2 (2)
C9—N2—C13—C18	112.19 (17)	C38—C37—C42—C41	2.7 (2)
N3—N2—C13—C18	−32.53 (19)	C43—C37—C42—C41	−173.10 (15)
C9—N2—C13—C14	−65.07 (19)	C44—N7—C43—C37	172.49 (13)
N3—N2—C13—C14	150.21 (13)	C38—C37—C43—N7	−162.46 (14)
C18—C13—C14—C15	−0.4 (2)	C42—C37—C43—N7	13.3 (2)
N2—C13—C14—C15	176.85 (13)	C43—N7—C44—C46	170.79 (14)
C13—C14—C15—C16	0.5 (2)	C43—N7—C44—C45	−19.0 (2)
C14—C15—C16—C17	0.2 (2)	N9—N8—C45—O6	−170.23 (13)
C15—C16—C17—C18	−0.9 (2)	C49—N8—C45—O6	−15.3 (2)
C14—C13—C18—C17	−0.3 (2)	N9—N8—C45—C44	6.01 (15)
N2—C13—C18—C17	−177.52 (13)	C49—N8—C45—C44	160.93 (13)
C16—C17—C18—C13	1.0 (2)	C46—C44—C45—O6	172.67 (15)
C27—N5—N6—C28	5.40 (15)	N7—C44—C45—O6	1.2 (3)
C31—N5—N6—C28	158.95 (13)	C46—C44—C45—N8	−3.10 (16)
C27—N5—N6—C29	156.14 (12)	N7—C44—C45—N8	−174.53 (14)
C31—N5—N6—C29	−50.31 (17)	N8—N9—C46—C44	4.76 (16)
C24—C19—C20—C21	0.5 (2)	C47—N9—C46—C44	151.81 (14)
C25—C19—C20—C21	−178.02 (13)	N8—N9—C46—C48	−174.57 (13)
C19—C20—C21—C22	0.5 (2)	C47—N9—C46—C48	−27.5 (2)
C20—C21—C22—O3	177.13 (13)	N7—C44—C46—N9	170.93 (13)
C20—C21—C22—C23	−1.4 (2)	C45—C44—C46—N9	−1.03 (16)
O3—C22—C23—C24	−177.20 (13)	N7—C44—C46—C48	−9.8 (2)
C21—C22—C23—C24	1.2 (2)	C45—C44—C46—C48	178.24 (14)
C22—C23—C24—C19	−0.2 (2)	C45—N8—C49—C54	−119.28 (16)
C20—C19—C24—C23	−0.6 (2)	N9—N8—C49—C54	32.9 (2)
C25—C19—C24—C23	177.84 (14)	C45—N8—C49—C50	59.9 (2)
C26—N4—C25—C19	−177.08 (13)	N9—N8—C49—C50	−147.93 (14)
C24—C19—C25—N4	1.1 (2)	C54—C49—C50—C51	0.4 (2)
C20—C19—C25—N4	179.49 (14)	N8—C49—C50—C51	−178.77 (14)
C25—N4—C26—C28	−158.83 (14)	C49—C50—C51—C52	−1.4 (3)
C25—N4—C26—C27	27.2 (2)	C50—C51—C52—C53	1.0 (3)
N6—N5—C27—O4	172.41 (12)	C51—C52—C53—C54	0.5 (3)
C31—N5—C27—O4	20.4 (2)	C50—C49—C54—C53	1.1 (2)
N6—N5—C27—C26	−5.35 (15)	N8—C49—C54—C53	−179.82 (14)
C31—N5—C27—C26	−157.38 (13)	C52—C53—C54—C49	−1.5 (2)
C28—C26—C27—O4	−174.11 (15)		

### Hydrogen-bond geometry (Å, º)

**Table:**

*D*—H···*A*	*D*—H	H···*A*	*D*···*A*	*D*—H···*A*
O1—H1···O4	0.84	1.86	2.6947 (14)	170
O3—H3*A*···O6^i^	0.84	1.86	2.6993 (15)	175
O5—H5*A*···O4	0.84	1.88	2.7218 (15)	179
C7—H7···O2	0.95	2.30	3.009 (2)	131
C25—H25···O4	0.95	2.46	3.0612 (18)	121
C43—H43···O6	0.95	2.36	2.9989 (18)	124
C33—H33···O6^ii^	0.95	2.42	3.168 (2)	135
C35—H35···O2^ii^	0.95	2.31	3.122 (2)	143

Symmetry codes: (i) *x*−1, *y*, *z*; (ii) *x*, *y*+1, *z*.
