# Supplementary figures and images for: Crystal structure of 4-[(E)-(4-hy­droxy­benzyl­idene)amino]-1,5-dimethyl-2-phenyl-1H-pyrazol-3(2H)-one
Source: Acta Crystallogr E Crystallogr Commun. 2015 Nov 14;71(Pt 12):o947–8. doi: 10.1107/S2056989015021325 (PMC4719916; doi:10.1107/S2056989015021325)

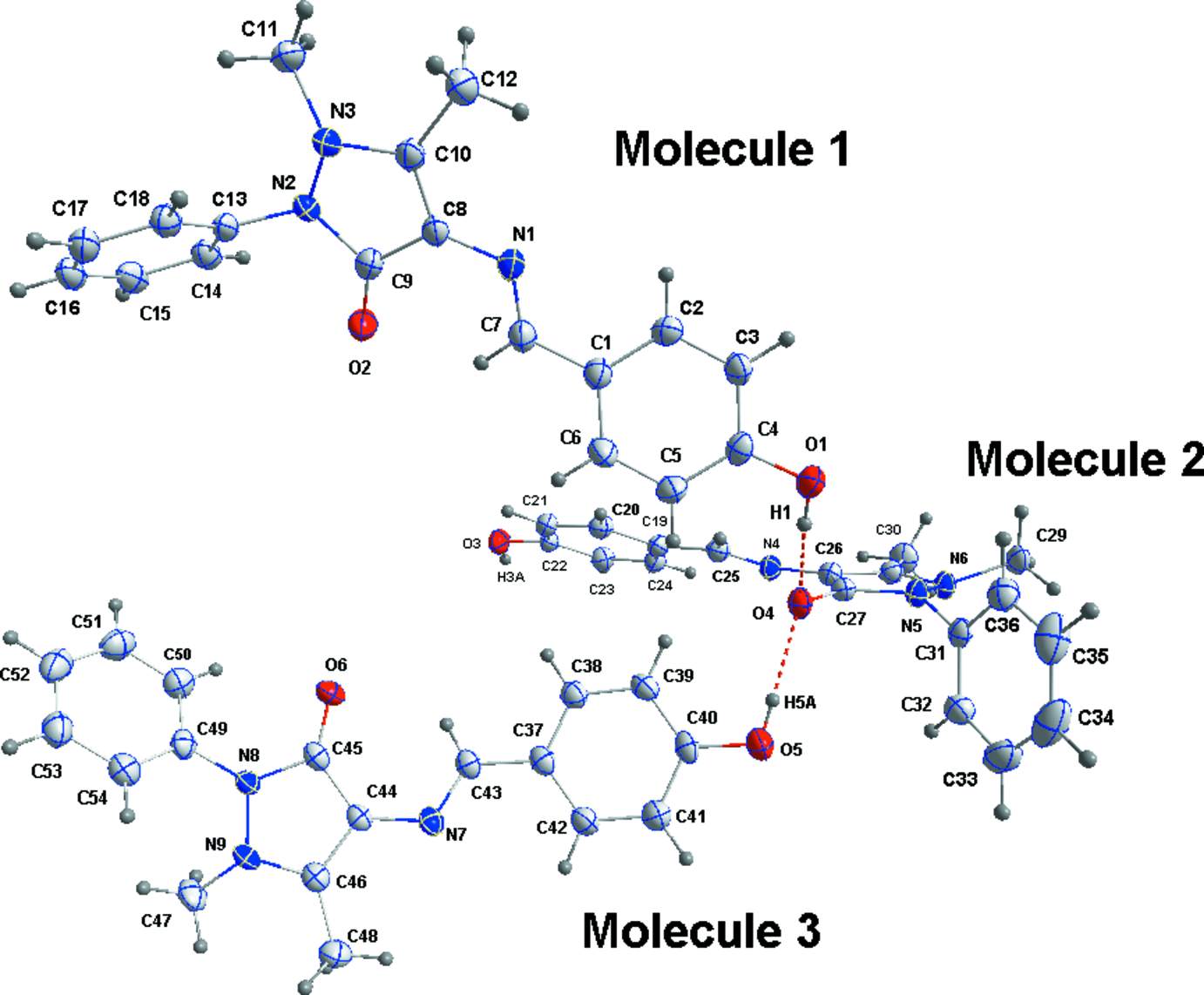

Supplement: Supplementary file 4 [file e-71-0o947-fig1.tif]

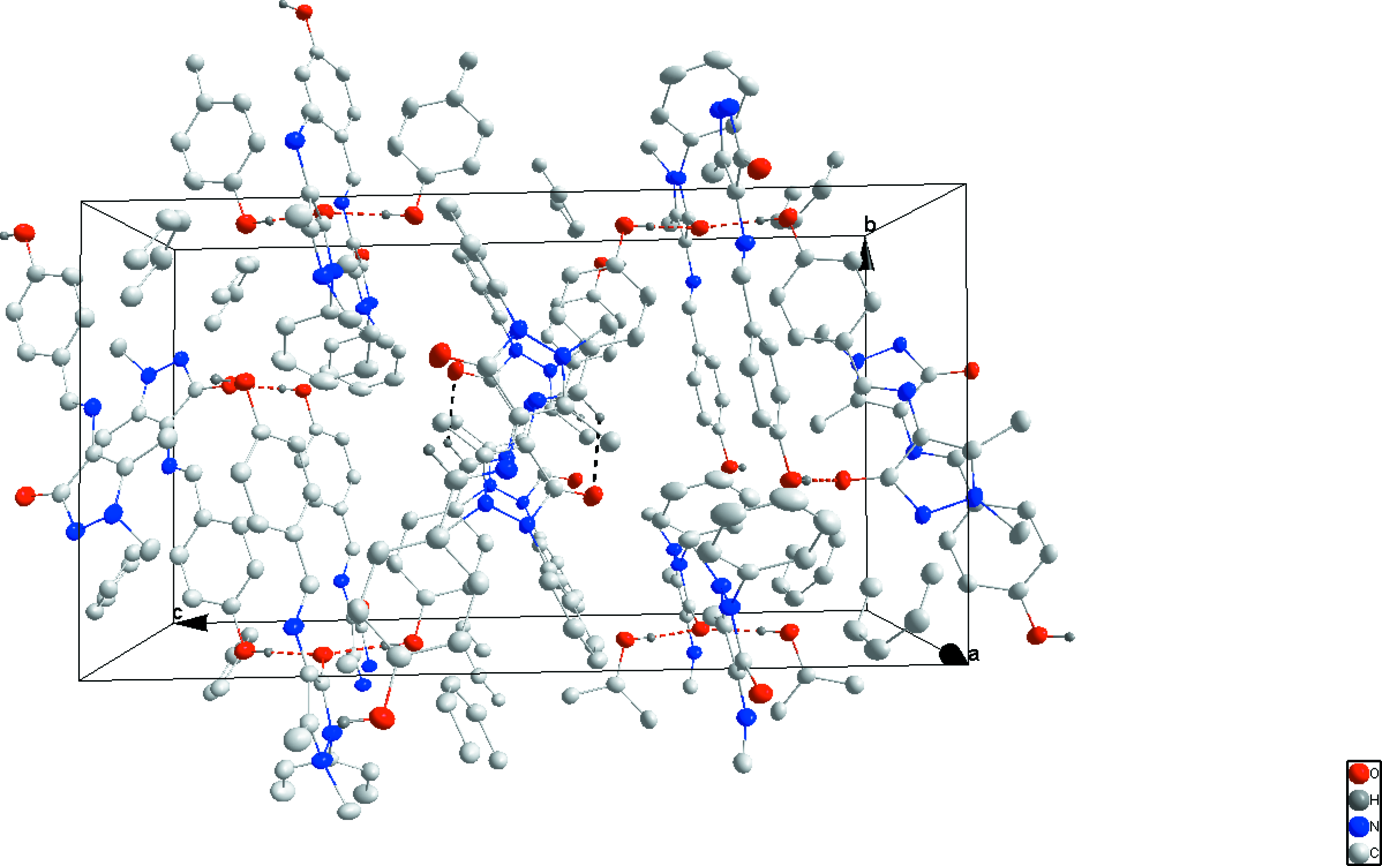

Supplement: Supplementary file 5 [file e-71-0o947-fig2.tif]
